# Do maternal albumin levels affect post-operative complications after cesarean delivery?

**DOI:** 10.1186/s12884-022-05215-8

**Published:** 2022-12-06

**Authors:** Yael Yagur, Rachel Ribak, Emili Ben Ezry, Ido Cohen, Libby Or Madar, Michal Kovo, Tal Biron-Shental

**Affiliations:** Department of Obstetrics and Gynecology, Meir Medical Center, Kfar Saba, Israel Affiliated With Sackler Faculty of Medicine, Tel Aviv University, Tel Aviv, Israel

**Keywords:** Serum Albumin, Cesarean Delivery (CD), Postoperative outcome, Surgical site infection (SSI)

## Abstract

**Background:**

This study explored the correlation between maternal serum albumin levels prior to elective cesarean delivery (CD) and postoperative complications.

**Methods:**

This retrospective cohort study included women admitted for elective CD at term to our tertiary referral center, during the years 2016–2018. Blood samples were collected during the preoperative admission. Information collected included maternal demographics, pregnancy and postoperative complications. Data between patients with preoperative serum albumin levels < 3.3 g/dL or ≥ 3.3 g/dL were compared.

**Results:**

Among 796 women admitted for an elective CD, 537 met the inclusion criteria. There were 250 (46.6%) women in the low albumin level group (< 3.3 g/dL) and 287 (53.4%) with serum albumin level ≥ 3.3 g/dL. Patients with serum albumin ≥ 3.3 g/dL had increased rates of surgical site infection (SSI) (5.6% vs. 1.6% respectively; *p* = 0.02), need for antibiotics during the post-partum period (10.8% vs 3.2%, respectively; *p* = 0.001), surgical intervention (2.1% vs. 0%, respectively; *p* = 0.03) and higher rate of rehospitalization (5.2% vs. 0.4%, respectively; *p* = 0.001). Multivariant analysis showed that albumin level ≥ 3.3 g/dL was independently associated with composite postoperative adverse maternal outcome.

**Conclusions:**

High serum albumin levels among women undergoing CD, might be associated with abnormal postoperative outcomes. Larger prospective studies, with a heterogenous population are needed to validate these observations.

## Introduction

Cesarean delivery (CD) is one of the most common surgical procedures performed worldwide. The rates have been increased throughout the years and are about 15–35% [1–3]. The major complications related to CD are infectious as surgical site infection (SSI) or endometritis. Other common complications include hemorrhage, injury to related organs and thromboembolic disorders [4]. The risk of maternal morbidity is higher after CD during labor. Endometritis has been reported in up to 6% of primary CD, performed before labor, as compared to 11% when CD performed during labor. Wound complications occur in up to 2% of primary CD and up to 28% in unplanned CD [3, 5–7].

It is well-established that post-operative complications are influenced by performance status prior to surgery [8], among other factors as fluid balance, anemia, and anesthetic technique [9–11]. Physiological changes during pregnancy might also impact maternal performance status, therefore, it is important to identify and characterize pre-operative risk-factors of pregnant women, to improve their outcomes.

Albumin is the main plasma protein in the body. The liver produces about 15 g of albumin per day (200 mg per kilogram of body weight). Its level in the serum reflects its rate of formation, decomposition and the volume of dispersion. Albumin levels are affected by nutritional status, oncotic pressure, cytokines and hormones [12]. Albumin levels are one of the diagnostic signs for pre-operative nutritional status. In recent years, several studies have shown correlations between hypoalbuminemia and postoperative mortality and morbidity in several types of surgeries. Hypoalbuminemia was found to be an independent risk factor for a surgical site infection [13–15]. However, these studies focused on oncology patients and surgeries involving the gastrointestinal tract.

During pregnancy, plasma volume increases by 50% and red blood cells by 30%; therefore, there is a physiological hemodilution [16]. This hemodilution leads to decreased serum albumin levels beginning in the first trimester, which becomes more accentuated with advanced gestation. “Normal” albumin values during pregnancy are not well-established [17]. The physiological pregnancy-related hemodilution may also influence the immune status and the bioavailability of drugs such as antibiotics, which are given before every CD [18].

Studies attempted to establish associations between serum albumin and obstetrical complications are scarce. It was demonstrated that decline in serum albumin levels could predict the development of preeclampsia [19], while another study showed an association between hyperalbuminemia and development of fetal growth restriction [20].

To the best of our knowledge, the correlation between maternal albumin levels and post-CD complications has not been investigated. Therefore, we aimed to fill this gap, hypothesizing that low albumin levels will correlate with adverse post-CD maternal outcome.

## Methods

### Patients

This retrospective cohort study included pregnant women admitted for an elective CD at term (> 37 weeks of gestation) to the Obstetrics and Gynecology department at Meir Medical Center, during the years 2016–2018. The study was approved by the local Institutional Review Board, #038–16.

Indications for CD included fetal malpresentation, abnormal placentation, macrosomia (defined as estimated fetal weight > 4000 gr in patients with diabetes mellitus (DM) and gestational DM or > 4500 gr in healthy pregnant women) [21], previous CD or other uterine surgery [22].

**Fig. 1:**
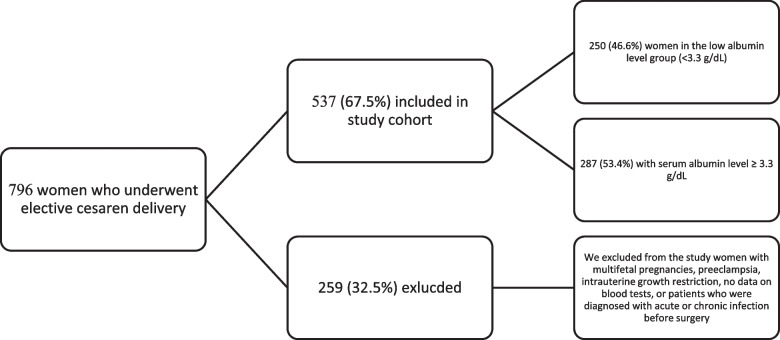
Study flow diagram. Figure Legend: This flow diagram demonstrating the cohort that included for analysis. We included 796 women for analysis who admitted for elective cesarean delivery. Only 537 were include in the final cohort. We excluded from the study women with multifetal pregnancies, preeclampsia, intrauterine growth restriction, no data on blood tests, or patients who were diagnosed with acute or chronic infection before surgery

Excluded from the study women with multifetal pregnancies, preeclampsia, intrauterine growth restriction, no data on blood tests, or patients who were diagnosed with acute or chronic infection before surgery (Fig. 1).

All study participants were given IV prophylactic antibiotics before the CD (cefazolin based on maternal weight, 2 g for those up to 80 kg and 3 g for patients weighing above 80 kg), according to departmental protocol as recommended [23].

### Blood samples

Maternal blood samples were taken during the preoperative admission, a day before surgery. Written informed consent was not required, due to the nature of the study and approved by institutional review board. The samples were collected and transported to a central laboratory for analysis, including albumin levels, complete blood count and chemistry. The albumin and chemistry were analyzed using an AU5800 system (Beckman Coulter, Brea, CA, USA). Complete blood count was analyzed with an ADVIA 2120i (Siemens AG, Germany). Reference for plasma albumin was 3.5–4.5 g/dL and total protein 6.0–8.2 g/dL.

### Data

Data were collected from electronic medical records, including maternal demographics, pregnancy complications, and postoperative complications (maternal fever (defined as > 38 °C after the first 24 h postpartum), surgical site infection (SSI), intra-abdominal hematoma, need for relaparotomy, hospitalization days and rehospitalization after discharge.

Since there are no albumin levels nomograms for pregnant women, we calculated the quartiles, of albumin levels, of the studied cohort, and found that the mean and median of albumin level was 3.3 g/dL. We generated a ROC curve and found that the most discriminatory cutoff for our population was 3.3 g/dL. Comparison of maternal characteristics and outcomes was performed between patients with preoperative serum albumin levels < 3.3 g/dL and ≥ 3.3 g/dL.

The datasets generated during and/or analyzed during the current study are available in the corresponding author repository.

### Ethics approval and consent to participate

This study was performed in line with the principles of the Declaration of Helsinki. All methods were carried out in accordance with relevant guidelines and regulations. All experimental protocols were approved by Meir Medical Center Human Investigation Committee number MMC-0038–16. Due to the retrospective nature of the study, Meir Medical Center Human Investigation Committee has waived the need for informed consent statement.

### Statistical analysis

The rate of surgical wound infection after elective CD is about 5% [24]. Power analysis revealed that a sample size of 432 patients would be sufficient to detect a 10% difference in parameters that could predict double the composite maternal postoperative complications, under the assumptions of a type I error (two-sided) of 5% and at least 80% power.

Composite adverse post-operative maternal outcome was defined as the presence of one or more of the following complications: surgical site infection (SSI), need for antibiotics and rehospitalization. Rehospitalization included patients who hospitalized after discharged due to SSI and endometritis that needed an intravenous (IV) antibiotics treatment, or those who had wound complications.

Patients’ characteristics were compared between the control and the study groups, using student *t-test* for continuous variables, and the *chi-square* or *Fisher’s Exact Test* for categorical variables. Results were considered significant when the p-value was ≤ 0.05. Data are presented as numbers and percentages for categorical variables, and as means and standard deviations for continuous variables. All statistical analyses were performed using SPSS Statistics for Windows (IBM Corp., Armonk, NY).

A logistic regression was composed, in which the composite adverse post-operative maternal outcome served as the dependent variable, while maternal age, BMI, diabetes mellitus (DM), albumin level (≥ 3.3 g/dL), hematocrit, and cholesterol levels at admission, served as independent variables.

## Results

During the study period, 537 of 796 women who were admitted for an elective CD, met the inclusion criteria. Albumin levels ranged from 2.4 g/dL to 4.3 g/dL with mean and median values of 3.3 g/dL. There were 250 (46.6%) women in the low albumin level group (< 3.3 g/dL) and 287 (53.4%) with high albumin levels (≥ 3.3 g/dL).

Table 1 presents maternal and obstetric characteristics of the study groups. There were no between groups differences in maternal age, ethnicity, smoking, gravidity or maternal comorbidities, such as diabetes mellitus. Neonatal weight did not differ between the groups. As compared to women with albumin level < 3.3 g/dL, more patients with albumin ≥ 3.3 g/dL had BMI < 25 kg/m^2^ (*p* = 0.02).

**Table 1:** Demographic characteristics of the study groups

**Characteristic**	***Serum albumin level* < *3.3*** ***(n* = *250)***	***Serum albumin level* ≥ *3.3*** ***(n* = *287)***	***P-value***
Age (years)	34.4 ± 5.3	33.7 ± 5.3	0.15
Ethnicity			
Jewish	189 (74.4%)	229 (80.1%)	0.14
Muslim	63 (25.3%)	57 (19.9%)	
C*esarean delivery*			
No previous CD	66 (26.4%)	56 (19.5%)	0.11
One previous CD	98 (39.2%)	113 (39.4%)	
More than one previous CD	86 (34.4%)	118 (41.1%)	
Diabetes mellitus	40 (16.1%)	50 (17.7%)	0.62
Smoker	13 (5.3%)	18 (6.4%)	0.60
BMI (kg/m^2)^			
BMI < 25	130 (57%)	175 (67.3%)	0.02
BMI ≥ 25	98 (43%)	85 (32.7%)	
Primigravida	43 (17.2%)	29 (10.1%)	0.02
Laboratory tests at admission			
Hemoglobin (g/dl)	11.3 ± 1.2	11.8 ± 1.1	< 0.001
Hematocrit (%)	33.9 ± 3.9	35.2 ± 3.2	< 0.001
White blood cells (K/microL)	8.9 ± 2.3	9.4 ± 2.1	0.009
Platelets (K/microL)	207.1 ± 59	209.1 ± 53.6	0.68
Total protein (g/dl)	6.2 ± 0.4	6.8 ± 0.3	0.007
Cholesterol (mg/dL)	262.9 ± 55.1	279.5 ± 57	0.001
Neonatal birth-weight (g)	3149 ± 516	3197 ± 536	0.27

Data are shown as number (%), mean ± standard deviation or median (range), as appropriate; GDMA1-gestational diabetes mellitus A1; GDMA2-gestational diabetes mellitus A2; BMI-body mass index (kg/m^2^)

Additional laboratory tests differed between the groups. As compared to patients in the low albumin group (< 3.3 g/dl), the high albumin group (≥ 3.3 g/dL) was characterized by higher levels of hemoglobin (Hb, g/dL), *p* < 0.001, hematocrit (HCT, %), *p* < 0.001, white blood cell count (WBC, K/microL), *p* = 0.009, total protein (g/dL), *p* = 0.007 and cholesterol (mg/dL) *p* = 0.001.

Table 2 presents post-partum maternal morbidity of the study groups. As compared to women in the low albumin group(< 3.3 g/dL), patients in the high albumin group (≥ 3.3 g/dL) had increased rates of SSI, (5.6% vs. 1.6%, respectively; *p* = 0.02), antibiotic treatment during the post-partum period (10.8% vs. 3.2% respectively; *p* = 0.001), surgical intervention (2.1% vs. 0% respectively; *p* = 0.03) and rehospitalization (5.2% vs. 0.4%, respectively; *p* = 0.001).

**Table 2:** Post-partum maternal morbidity

***Variable***	***Serum albumin level* < *3.3*** ***(n* = *250)***	***Serum albumin level* ≥ *3.3*** ***(n* = *287)***	***P-value***
Duration of hospitalization (days)	3.1 ± 0.4	3.0 ± 0.3	0.08
Rehospitalization rate	1 (0.4%)	15 (5.2%)	0.001
Surgical site infection	4 (1.6%)	16 (5.6%)	0.02
Positive blood culture	1 (0.6%)	7 (3.4%)	0.05
Antibiotic treatment	8 (3.2%)	31 (10.8%)	0.001
Surgical intervention	0 (0%)	6 (2.1%)	0.03
Laboratory tests post operative			
Hemoglobin (g/dl)	10.4 ± 1.3	10.7 ± 1.3	0.003
White blood cells (K/microL)	10.1 ± 2.5	10.6 ± 2.5	0.04
Platelets (K/microL)	192.2 ± 55.2	194.8 ± 53.6	0.6
Need for blood transfusion	2 (0.8%)	3 (1.1%)	1

Data are shown as number (%), mean ± standard deviation or median (range), as appropriate

By multivariant logistic regression analysis we found that albumin level ≥ 3.3 g/dl was independently associated with the adverse composite maternal outcome, OR 3.75 (95%CI 1.65–8.49), *p* = 0.02; Table 3).

**Table 3:** Regression model for composite adverse postpartum maternal outcome

***Parameter***	***O*dds ratio *(95% CI)***	***P*-*value***
Age (years)	0.71 (0.28–1.79)	0.47
Smoker	1.45 (0.45–4.65)	0.53
BMI (Kg/m^2^)	0.77 (0.37–1.59)	0.48
DM	1.63 (0.73–3.62)	0.23
Albumin ≥ 3.3 g/dl	3.75 (1.65–8.49)	0.02
Hematocrit (%)	1.08 (0.97–1.19)	0.15
Cholesterol (mg/dL)	1.00 (0.99–1.19)	0.53

*CI* Confidence interval, Composite adverse maternal outcome was defined the presence of any of the following postpartum complications: surgical site infection, need for antibiotics and rehospitalization. *DM* Diabetes mellitus includes gestational and pre-gestational DM

## Discussion

The current study found an association between maternal postoperative adverse outcomes and serum albumin ≥ 3.3 g/dL, prior to CD. Specifically, higher rates of SSI, need for antibiotics in the post-partum period and rehospitalization after elective CD.

The association between plasma protein levels in pregnancy and adverse maternal or fetal outcomes is not well understood. Notably, there are no nomograms for serum albumin levels during pregnancy; yet, albumin is one of the plasma expander proteins. Plasma volume begins to increase from as early as the first weeks of pregnancy and continues to increase until term [25].

Albumin is known to be an important transport protein with antioxidant characteristics. Serum albumin levels during pregnancy might be affected by maternal inflammatory and malnutrition status or protein metabolism [26].

An association between low serum albumin and increased risk for postoperative complications was observed among adults with severe diseases, such as oncology patients, orthopedics and major operations, although the mechanism has not been fully elucidated. Additionally, very low serum albumin levels were also found to be predictors of mortality among extremely low birth weight infants [6, 14, 15, 27].

In the current study, we unexpectedly observed an increased odds ratio for composite adverse maternal postoperative outcome among women with serum albumin level ≥ 3.3 g/dL (OR 3.75, 95%CI 1.65–8.49, *p* = 0.02). On the other hand, others found an increased incidence of pre-eclampsia and hypertension in women with low serum albumin [18, 19]. It could be speculated that changes in serum albumin, whether increased or decreased from the normal rang, may represent abnormal volume expansion during pregnancy and therefore, may affect maternal pregnancy outcomes.

In the same line of thought, a recent study demonstrated a reverse U-shaped association between maternal serum albumin and fetal growth, with high levels of maternal serum albumin contributing to the development of fetal growth restriction [20].

Further research is warranted to investigate the mechanisms of the associations between maternal serum albumin and outcomes. Moreover, larger studies are needed to establish the findings before performing a routine pre-cesarean albumin levels measurements for every elective CD. Nevertheless, in any case data is available, clinicians should be aware of these findings, to optimize postoperative care to prevent possible severe maternal complications.

There are several strengths to the current study. First, to our knowledge it is the first study who investigate the correlation between maternal serum albumin level and post CD complications while adjusting for several confounders. Second, the study population was urban, with similar maternal characteristics.

The study is not without limitations. First, the relatively small sample limited us from studying maternal outcomes at other serum albumin levels. The study population included patients who underwent elective CD, with a high rate of gestational diabetes among the participants. Thus, the generalizability of the observed associations may be limited.

In conclusion, high serum albumin levels among women undergoing CD might be associated with adverse postoperative outcomes. Larger prospective studies, with a heterogenous population, are needed to validate these observations.

## Data Availability

The datasets used and/or analysed during the current study are available from the corresponding author on reasonable request.

## Abbreviations

CD: Cesarean Delivery
SSI: Surgical site infection
DM: Diabetes mellitus
