# Correction: Do maternal albumin levels affect post-operative complications after cesarean delivery?

**DOI:** 10.1186/s12884-022-05313-7

**Published:** 2022-12-31

**Authors:** Yael Yagur, Rachel Ribak, Emili Ben Ezry, Ido Cohen, Libby Or Madar, Michal Kovo, Tal Biro

**Affiliations:** Department of Obstetrics and Gynecology, Meir Medical Center, Kfar Saba, Israel Affiliated With Sackler Faculty of Medicine, Tel Aviv University, Tel Aviv, Israel

**Correction: BMC Pregnancy Childbirth 22, 909 (2022)**

**https://doi.org/10.1186/s12884-022-05215-8**

Following the publication of the original article [1], it was noted that due to a typesetting error the heading of the second column of Tables 1 and 2 should read “Serum albumin level < 3.3 (*n* = 250).

**Table 1:** Demographic characteristics of the study groups

Characteristic	Serum albumin level < 3.3 (***n*** = 250)	Serum albumin level ≥ 3.3 (***n*** = 287)	***P***-value
Age (years)	34.4 ± 5.3	33.7 ± 5.3	0.15
Ethnicity			
Jewish	189 (74.4%)	229 (80.1%)	0.14
Muslim	63 (25.3%)	57 (19.9%)	
Cesarean delivery			
No previous CD	66 (26.4%)	56 (19.5%)	
One previous CD	98 (39.2%)	113 (39.4%)	0.11
More than one previous CD	86 (34.4%)	118 (41.1%)	
Diabetes mellitus	40 (16.1%)	50 (17.7%)	0.62
Smoker	13 (5.3%)	18 (6.4%)	0.60
BMI (kg/m^2)^			
BMI < 25	130 (57%)	175 (67.3%)	0.02
BMI ≥25	98 (43%)	85 (32.7%)	
Primigravida	43 (17.2%)	29 (10.1%)	0.02
Laboratory tests at admission			
Hemoglobin (g/dl)	11.3 ± 1.2	11.8 ± 1.1	< 0.001
Hematocrit (%)	33.9 ± 3.9	35.2 ± 3.2	< 0.001
White blood cells (K/microL)	8.9 ± 2.3	9.4 ± 2.1	0.009
Platelets (K/microL)	207.1 ± 59	209.1 ± 53.6	0.68
Total protein (g/dl)	6.2 ± 0.4	6.8 ± 0.3	0.007
Cholesterol (mg/dL)	262.9 ± 55.1	279.5 ± 57	0.001
Neonatal birth-weight (g)	3149 ± 516	3197 ± 536	0.27

Data are shown as number (%), mean ± standard deviation or median (range), as appropriate; GDMA1-gestational diabetes mellitus A1; GDMA2-gestational diabetes mellitus A2; BMI-body mass index (kg/m^2^)

**Table 2:** Post-partum maternal morbidity

Variable	Serum albumin level < 3.3 (***n*** = 250)	Serum albumin level ≥ 3.3 (***n*** = 287)	***P***-value
Duration of hospitalization (days)	3.1 ± 0.4	3.0 ± 0.3	0.08
Rehospitalization rate	1 (0.4%)	15 (5.2%)	0.001
Surgical site infection	4 (1.6%)	16 (5.6%)	0.02
Positive blood culture	1 (0.6%)	7 (3.4%)	0.05
Antibiotic treatment	8 (3.2%)	31 (10.8%)	0.001
Surgical intervention	0 (0%)	6 (2.1%)	0.03
Laboratory tests post operative			
Hemoglobin (g/dl)	10.4 ± 1.3	10.7 ± 1.3	0.003
White blood cells (K/microL)	10.1 ± 2.5	10.6 ± 2.5	0.04
Platelets (K/microL)	192.2 ± 55.2	194.8 ± 53.6	0.6
Need for blood transfusion	2 (0.8%)	3 (1.1%)	1

Data are shown as number (%), mean ± standard deviation or median (range), as appropriate

The correct tables have been included in this correction, and the original article has been corrected.
